# Managing the morass. Lessons learned from establishing a data linkage model for long-term follow up of a trial cohort using routine health and education data

**DOI:** 10.1186/1745-6215-16-S2-O70

**Published:** 2015-11-16

**Authors:** Fiona Lugg, Rebecca Cannings-John, Gwenllian Moody, Mike Robling

**Affiliations:** 1 Cardiff University, Cardiff, UK

## 

The Building Blocks trial [ISRCTN 23019866] assessed the short-term impact of an intensive programme of antenatal and postnatal visiting by specially trained nurses to support young pregnant women. This follow-on study, BB: 2-6, will assess the programmes' impact on longer-term benefits for mothers and children, through the linkage of routinely collected data, with a particular emphasis on the programmes' impact upon child maltreatment.

Follow up will be by linked anonymous data abstraction from the Health and Social Care Information Centre (HSCIC), Office for National Statistics (ONS) and Department for Education, National Pupil Database (NPD). These information centres (ICs) will match participants to the information held in their databases, and send to a third party safe haven.

Re-consenting of all mothers would have resulted in a drastic loss of participants therefore section 251 support was sought to allow the transfer of identifiers to ICs for matching. This support required participants to opt-out rather than consent in.

This project will complete in 2018. What has been achieved thus far is the establishment of a regulatory compliant model of linking health, social care and education data to clinical data. This model of linkage offers the possibility for long term evaluation of trials, at lower cost, and with the potential to extend to other sources of routine data.

This presentation will review the approach established in this study, reflect on the dis/advantages of the approach, summarise the conditions of s251 support, information governance compliance, IC requirements and finally the challenges (and solutions) faced thus far.

